# The impact of virtual reality meditation on college students’ exam performance

**DOI:** 10.1186/s40561-021-00166-7

**Published:** 2021-10-16

**Authors:** Regina Kaplan-Rakowski, Karen R. Johnson, Tomasz Wojdynski

**Affiliations:** 1 Department of Learning Technologies, College of Information, University of North Texas, 3940 N. Elm Street, G159, Denton, TX 76207 USA; 2 Department of Learning Technologies, College of Information, University of North Texas, 3940 N. Elm Street, G150, Denton, TX 76207 USA; 3 School of Banking and Management in Cracow, Aleja Kijowska 14, 30-079 Kraków, Poland

**Keywords:** Mindfulness, Meditation, Virtual reality, Anxiety, Well-being, Exam

## Abstract

Advocates of meditation claim that it can improve various aspects of life, including health, attention, thinking, and learning. The purpose of this empirical, quantitative, between-subject study was twofold. First, it compared the effectiveness of meditation delivered through virtual reality versus video, as measured by students’ test scores. Second, the study provided insights on the use of meditation, whether via virtual reality or video, as a way to positively affect well-being. *T*-test analysis showed virtual reality meditation to be significantly more beneficial than video meditation. Students reported that meditation techniques delivered using either medium to be helpful in decreasing their pre-exam anxiety. This study has practical implications and offers evidence on the beneficial impact of VR meditation on students’ exam performance and anxiety levels.

## Introduction

Meditation practices are becoming more widespread in Western societies because of their positive impact on the brain and mind (Tang et al., 2015). Meditation has been found to have therapeutic benefits for a variety of mental and physical conditions, such as anxiety, depression, stress, insomnia, and post-traumatic stress disorder (Davidson & Kaszniak, 2015; Shamekhi & Bickmore, 2015). Meditation is also associated with mindfulness, which is a contemporary secular adaptation of traditional meditation practices with the goal of improving cognitive focus (Hyland, 2015). Our study is concerned with the contemporary variation of meditation as a relaxation technique to aid students.

The application of meditation in Western societies has been expanding beyond the field of medicine and health care to the workplace (Johnson & Park, 2020; Johnson et al., 2020). As an approach to enhance learning, meditation is being slowly infused into educational settings (Napora, 2011; Yun et al., 2020) and has been explored in relation to student anxiety, stress, depression, optimism, and overall health (Waters et al., 2015). Meditation practices are used in schools (Levete, 2001) and in online settings (Palalas et al., 2020) to promote cognitive and affective development.

While meditation has existed to improve health and well-being for millennia, two recent developments motivate a reexamination of how it may aid educational practices. First, technological progress, especially in virtual reality (VR) technology, has provided new forms of multimedia content delivery that may be particularly well suited as meditational aids. Such advances could be particularly useful to those populations who lack access to traditional guides or content on meditation practices. Second, meditation as a relaxation technique is potentially more vital today because of the increased interest in supporting students’ emotional needs triggered by the COVID-19 pandemic, which exacerbated students’ mental health issues, stress, and anxiety (Kaplan-Rakowski, 2020; Kecojevic et al., 2020; Son et al., 2020). In 2020, over 50% of college students reported increased stress levels associated with the pandemic (American College Health Association, 2020).

Even though meditation is traditionally not associated with the use of technology, technology-delivered approaches are increasingly common for content that is potentially useful for meditation (Buie & Blythe, 2013a, 2013b). According to Shaw et al. (2011), meditation is enhanced and better supported when delivered with technology. Even music alone can have therapeutic and stress-reducing capabilities (Sudheesh & Joseph, 2000). Meditation can also be delivered using web-based videos (Davis et al., 2015), where calming music is enriched with visual stimuli. VR represents a potentially powerful tool to aid in meditation due to its affordances in terms of immersion, the sense of presence, and the suppression of external stimuli. In VR, in addition to music, users benefit from immersive computer-generated visualizations, allowing them to teleport elsewhere (e.g., to a relatively stress-free setting such as a forest or a beach) and, consequently, facilitating meditation practices (Navarro-Haro et al., 2017).

In fact, VR has been successfully used for treating phobias, post-traumatic stress (Maples-Keller et al., 2017), and anxiety (Rothbaum & Hodges, 1999). Given the ability of VR to induce relaxation states (Pizzoli et al., 2019; Riches et al., 2021) and knowing that relaxed states help with cognitive processing, we expect VR meditation to be beneficial in educational settings. More specifically, our aim is to address students’ mental health and well-being by exploring technology-mediated meditation as a technique that could positively impact students, especially prior to exams, which are traditionally associated with higher stress and anxiety.

## Literature review

To explore how VR- and video-based meditation could impact learners’ performance and well-being, we begin by providing an overview of literature on the practice of meditation in academic settings. We then explain two different vehicles with which meditation could be practiced, namely, VR and video.

### Meditation in academic settings

College students have generally reported feeling overwhelmed with constant pressures to keep pace with academic work (Sax, 1997). End-term or high-stakes exams are particularly stressful as students’ educational and future career opportunities depend on one-day performances (Rosiek et al., 2016). As evidenced in student surveys, exam pressure is likely to trigger stress, depression, and even suicidal thoughts (Rosiek et al., 2016). Although exam-specific pressure is not directly related to dramatic events such as the pandemic, the abrupt switch from face-to-face to online learning in 2020 (Ferdig et al., 2020; Hartshorne et al., 2020), economic hardship, health concerns, grief, and elevated levels of anxiety have put an additional toll on students’ mental health. Therefore, addressing students’ emotional needs has become more important than ever before (Kaplan-Rakowski, 2020). One way to foster students’ well-being is through introducing meditation practices that can reduce stress levels and improve academic performance (Kauts & Sharma, 2009).

When practiced by students, meditation enhances metacognitive skills and helps to foster critical, analytical, and creative thinking (Napora, 2011), attention (Valentine & Sweet, 1999), problem-solving abilities (Raingruber & Robinson, 2007), emotional intelligence (Rosaen & Benn, 2006), psychosocial strengths, and self-regulation (Wisner et al., 2010). These skills are needed to enhance classroom performance, including exam taking.

Studies by Mata (2012) and Ching et al. (2015) are two other examples of successful implementation of meditation in the classroom. Mata (2012) integrated a 5–7-min meditation to undergraduate students in an early childhood education course. Her results showed that meditation can calm, relax, and improve students’ centeredness, readiness for attention, concentration, and clarity of mind. Incorporating meditation techniques into a college curriculum can be a practical way to improve cognition, clarity of mind, and attention of college students (Ching et al., 2015; Mata, 2012). Further, Ching et al. (2015) found, through a one-semester mindfulness meditation course, increased improvement in attention and memory components of cognitive performance among university students. The students in the intervention group significantly advanced  their accuracy of digital vigilance task, choice reaction time, and spatial working memory.

### Virtual reality and meditation

The rapidly evolving VR technology has been increasingly recognized as having a large potential for learning (e.g., Pathan et al., 2020). The main reason is the ability of VR to provide simulations that resemble real-life experiences (Freina & Ott, 2015). Levels of immersion into VR content can vary, and they can be dependent on the content itself or the type of VR equipment used, or both (Hayes et al., 2021). Kaplan-Rakowski and Gruber (2019) defined high-immersion VR as “a computer-generated 360° virtual space that can be perceived as being spatially realistic, due to the high immersion afforded by a head-mounted device” (p. 552). In other words, users wearing a head-mounted device (sometimes called a headset) can look in either direction and view their virtual surroundings in a similar way as in real life. Research repeatedly confirms that VR provides the sense of presence or being “there” (Gruber & Kaplan-Rakowski, 2020; Kaplan-Rakowski & Gruber, 2021; Navarro-Haro et al., 2017).

The term *immersion* relates to the feeling of being physically present in a nonphysical world and is often associated with VR (Freina & Ott, 2015). Spatial immersion in VR, with limited interruptions and disturbances from the real world, promotes an increased sense of presence, making VR conducive to facilitate and practice meditation (Navarro-Haro et al., 2017). High-immersion VR is a beneficial platform to manage attention (Hoffman et al., 2001). As VR has become more affordable and accessible, the technology is being widely used in education and training to motivate students and enhance the learning process (Freina & Ott, 2015; Kaplan-Rakowski & Wojdynski, 2018). Although still in its infancy, VR meditation is used as a tool in educational and training settings to motivate students in the learning process.

As Navarro-Haro et al. (2017) pointed out, initial research on the implementation of VR-based mindfulness meditation revealed positive outcomes. In their pilot study, using Oculus Rift, participants experienced and floated down a calm three-dimensional computer-generated virtual river scene while listening to VR Dialectical Behavioral Therapy mindfulness meditation skills training. Participants showed high acceptance of VR as a technique to practice mindfulness meditation, by reporting moderate to high presence and being significantly more relaxed with reduced feelings of anxiety and sadness during the experience.

Kosunen et al. (2016) tested the neuroadaptive meditation system RelaWorld, which is a system accompanied by neurofeedback. That is, users’ brain activity was monitored with an electroencephalogram, allowing researchers to calculate the level of relaxation, focus, and presence when meditating via VR. The analysis revealed that using high-immersion VR (with a headset) was more effective in deepening the levels of meditation compared with when meditation was practiced via a desktop monitor (without a headset). More recently, Waller et al. (2021) found that 360° video-guided meditation using a VR headset increased college students’ sense of psychological and perceptual presence of the instructor when compared with meditation via the 2D standard desktop monitor. In an experimental study of undergraduate students, VR guided meditation generated greater positive affect with an experience of higher immersiveness and egocentricity versus the non-VR guided meditation (Miller et al., 2021).

### Video-based meditation

Apart from VR, video technology can also deliver meditation. In a pilot study, users were more receptive to a digital meditation coach when compared with a self-help video. Through interactivity with the digital meditation coach, participants’ anxiety was reduced, simultaneously improving levels of mindfulness (Shamekhi & Bickmore, 2015). Video-based meditation on its own shows that mindfulness meditation training yielded reasonable participant engagement on the intervention practice of web-based video with phone support (Davis et al., 2015).

In another study, brief video-based mindfulness program was implemented for physicians in lieu of the traditional mindfulness stress reduction program that required intensive time commitment. The aim of the video-based program was to teach deep breathing techniques to encourage mindfulness (Pflugeisen et al., 2016). Following an 8-week intervention, the findings indicated that video-based mindfulness program was effective to reduce stress and improve mindfulness skills in physicians (Pflugeisen et al., 2016).

Research indicates that video-based meditation promotes user engagement (Davis et al., 2015), but it was not as effective at reducing anxiety when compared with meditation conducted by a virtual agent (Shamekhi & Bickmore, 2015). Also, when meditation is practiced via VR, participants can relax and experience less anxiety and sadness (Navarro-Haro et al., 2017). When compared with meditation on a desktop monitor, VR-based meditation tends to be more effective (Kosunen et al., 2016). On the contrary, findings from Crosswell and Yun’s (2020) study on virtual meditation as a stress management strategy for students on college campuses revealed that lower heart rate and reduced stress levels were more attributable to sound or self-guided meditation when compared to VR meditation.

### The study rationale

Previous literature repeatedly shows that stress can be reduced with meditation (Navarro-Haro et al., 2017; Tang et al., 2015; Waters et al., 2015) which can be successfully used in the classroom (Ching et al., 2015; Mata, 2012), but the effect of meditation may depend on the type of technology with which meditation is delivered. Studies on meditation using VR (e.g., Kosunen et al., 2016; Navarro-Haro et al., 2017) indicate that VR technology tends to have a relaxing impact on users. Similarly, meditation using video technology (Davis et al., 2015; Pflugeisen et al., 2016) can be beneficial for relaxation. No previous studies to our knowledge compared which (VR vs video) technology can deliver meditation more effectively. Moreover, few existing studies on using VR or video technology for meditation were conducted in academic settings.

Since college students are usually hesitant to seek counselling or professional help from mental health professionals, then the implementation of interventions that proactively address well-being is essential (Crosswell & Yun, 2020). Prompted by previous research findings and given the importance of addressing students’ mental health and well-being (Kaplan-Rakowski, 2020), we filled the gap in literature by exploring VR- and video-based meditation in an academic setting to test which technology can better benefit students.

Videos are more accessible, more affordable, and more frequently used compared with VR, so we treated video-based meditation as the control condition. VR provides the immersive aspect that videos relatively lack. Therefore, VR-based meditation was our experimental condition. Our main research question is: What is the difference between VR meditation and video meditation in their impact on students’ test scores? We hypothesized that VR-based meditation would be more effective than video-based meditation. In addition, based on a follow-up survey, the study provided insights on the use of meditation, whether VR or video, as a way to positively affect college students’ exam performance and their anxiety states.

## Methods

### Participants

The sample consisted of 61 adult, European-based, university business students. As seen in Table 1, female volunteers (*n* = 35) constituted 57% of the sample, and male volunteers (*n* = 26) constituted 43% of the sample. Gender differences did not emerge. The average age of the participants was 20.89 (median = 20). The sample represented students taking an introductory computer science course. The study was incorporated into the regular class time and involved students that were willing to participate in the meditation activity. The participants’ course average was 4.07 (out of 5). The number of participants who reported having prior experience of meditation was 11 (18% of the sample). The students’ self-reported stress level before the exam was average. Following ethical rules for research involving human subjects, the researchers made the data and the participants unidentifiable.

**Table 1:** Demographic Information about the Participants

	*N*	Gender		Meditation experience	Age (mean)
		Female	Male		
Total	61	35	26	11	20.89
%		57	43	18	

The contextual setting was Poland, a particularly attractive location for this study because Poland is commonly viewed as a traditional conservative Christian country, which presumably has not shown to have a deep awareness of Buddhist-derived practices (Charzyńska & Heszen-Celińska, 2020). Meditation is not a long-established practice in Poland, compared with, for example, the United Kingdom, with historical ties to India and large immigrant communities, which make for a very different awareness of meditation in the culture. However, a more contemporary variation of meditation currently may be trendy in Poland.

### Study design, setting, and procedure

This empirical, between-subject study employed a pretest–posttest design with the participants being randomly assigned to two groups (Isaac & Michael, 1997). No learning activities took place between the pretest and the posttest, implying that the pretest–posttest difference should be close to zero, on average.

The sample (*N* = 61) was randomly assigned to two groups: a VR treatment group (*n* = 31) and a video control group (*n* = 30). Based on pretest scores, Levene’s two-sample *F*-Test for variances was used to confirm homogeneity of the groups prior to the treatment. The Levene *F*-statistic was insignificant (*p* = 0.15), confirming that the group assignments were appropriate for the study.

The questions on the pretest were randomly chosen from the same databank of questions that were used on the posttest (i.e., the final exam). The participants in the two groups were of similar average age and course grade. The self-reported levels of general anxiety of the students in the two groups were also comparable.

The procedure started with a briefing intended to inform the students about the five steps of the study: (1) pretest, (2) intervention (either VR or video meditation), (3) posttest (i.e., final exam), (4) follow-up survey, and (5) a demographic questionnaire. The experiment took place in a computer laboratory with students working individually.

### Intervention description

**Fig. 1:**
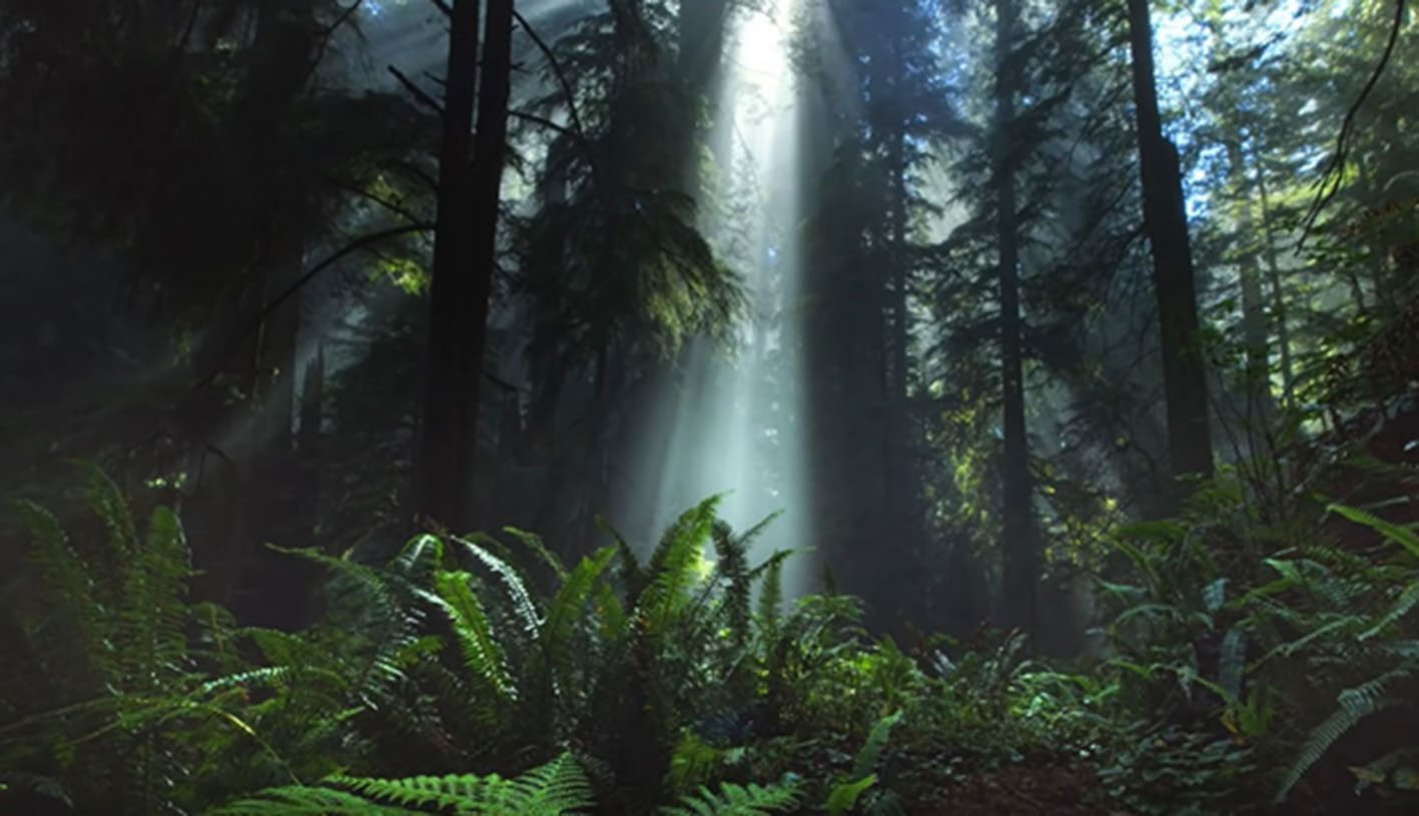
Screenshot of the Meditation Visualization

The interventions of the study consisted of two types of meditation: a VR-based meditation and a video-based meditation. Both interventions were comparable, with the main difference being the medium in which the meditation was practiced (VR versus video). The VR and video interventions both displayed animated, slow-paced, calming visualizations of forest scenes (see screenshot in Fig. 1). These visualizations were paired with music, which was also comparable across groups, with the same tempo (approximately 60 beats per minute) throughout the duration of the 15-min meditation sessions.

Studies into the differences between meditation of long and short durations have reached contradictory conclusions. Traditional belief was that meditation had to be practiced daily and for long periods (Fabio & Towey, 2018). A shortened version of meditation practice was considered ineffective. Meanwhile, some research confirms the benefits of short-term meditation because of its convenience and time efficiency (Kosunen et al., 2016; Tang et al., 2007). Our rationale for implementing short-term meditation was threefold. First, according to Tang et al. (2007), short-term meditation could be not only effective but also efficient. Especially in a synchronous classroom setting, taking time for a lengthy meditation is undesirable. Second, using VR for long periods of time is not recommended (Bailenson, 2018). Third, putting students in extended meditative states may induce sleepiness.

**Fig. 2:**
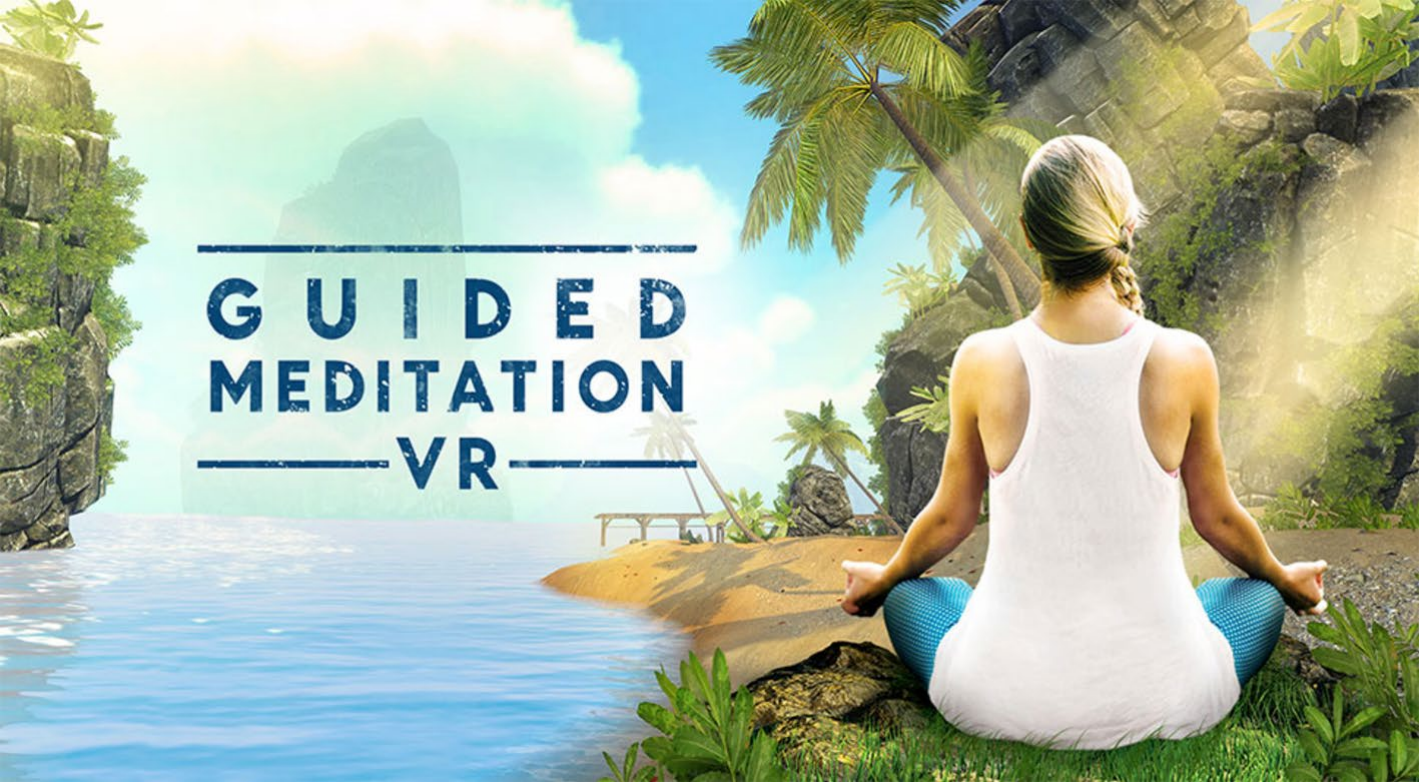
Screenshot of Guided Meditation VR App

The VR meditation took place using an Oculus Go headset with which subjects meditated using the freely available application called Guided Meditation VR (http://guidedvr.com/). Figure 2 displays the screenshot of the app. Meanwhile, the video clip was streamed from YouTube Channel (https://www.youtube.com/watch?v=hlWiI4xVXKY) and students watched it on a 17-inch desktop computer monitor. To facilitate the immersion of the meditation and to avoid distractions, the participants in both interventions used noise-canceling headphones.

### Instruments

The data collection took place in the same classroom as all the other study activities. The participants completed a pretest to verify the homogeneity between the groups. Next, participants were randomly assigned to the conditions using VR or video meditation. After the intervention, the students proceeded with the posttest (i.e., the final exam), a follow-up survey, and a demographic questionnaire.

To test whether any differences existed between the effect of VR and video meditation on students’ test performance, the scores received on the posttest served as the dependent variable. Performing *T-*tests, we calculated differences between the pretest–posttest scores of students who participated in the VR meditation and the video meditation.

The pretest and the posttest had the time limit set to 30 min, during that time the students were asked to perform basic computer science tasks involving the application of operational systems. The tasks were procedural, requiring students to follow a series of steps, for example: (1) creating a folder, (2) copying items to a folder, (3) creating a title page, (4) tracking changes, (5) renaming files, (6) compressing files, and (7) changing extensions.

The tests were multi-item, with total scores converted to percentages. Concerns about reliability and validity were solved by the implementation of previously validated tests. The test tasks/questions came directly from the question database of the course textbook, and they were of comparable difficulty. The scoring was straightforward. If the item was complete and correct, the students received full points. If the item was incomplete or incorrect, the student received zero points. No partial credit was given. The scoring followed the standards of the Pearson VUE Computer Based Test Development Center (https://home.pearsonvue.com/) for certificate exams.

The follow-up survey questions pertained to self-perceived anxiety levels, students’ relaxation practices, and general impressions of their meditation experience. The demographic questionnaire elicited information regarding students’ age, gender, electronic use, VR equipment ownership, and previous experience with VR.

## Findings

The aim of our study was to compare the effectiveness of meditation experienced through VR versus video, as measured by students’ test scores. We provide insights through student perceptions on the use of meditation, whether VR or video, as a way to positively affect college students’ anxiety states.

### The impact of VR versus video meditation

To examine whether the VR and the video groups differed in their prior mastery of computer science tasks, we conducted an independent samples *t* test based on pretest scores. The result of the two-sample independent *t* test comparing the differences in means between the two groups, assuming equal variances, was insignificant (*p* = 0.21). These calculations confirmed that on the pretest the VR and video groups were comparable.

We then compared the differences of pretest–posttest means between the VR and video groups. This approach is referred to as a difference in differences methodology in the wider social sciences and is considered a reliable method of inference when subjects are evaluated before and after a treatment effect (Donald & Lang, 2007). Table 2 displays data on pretest, posttest, and pretest–posttest differences. The most relevant results include the mean scores differences (0.03 for the VR group, − 0.19 for video group), standard deviation (0.27 for the VR group, 0.42 for the video group), degrees of freedom (59), the *p* value (0.01), *t*-statistic (2.41), and Cohen’s *d* (0.62). The value of 0.62 of the effect size is considered large.

**Table 2:** Results of Pretest, Posttest, and Pretest–Posttest Differences

	Intervention	*M*	*SD*	*df*	*t*	*p*	Cohen’s d
Pretest	VR	0.65	0.25		− 1.27	0.21	0.30
	Video	0.72	0.21	59			
Posttest	VR	0.67	0.27		1.66	0.05	0.42
	Video	0.53	0.39	59			
Pretest–posttest Differences	VR	0.03	0.27		2.41	0.01	0.62
	Video	− 0.19	0.42	59			

### Exploratory questions

In addition to investigating the impact of VR versus video meditation on students’ test scores, we asked the students six questions to uncover their perceptions of VR- and video-based meditation that they had before their exam.

One exploratory question was: “Do you think relaxation/meditation techniques could help you improve exam performance?” The majority of students (59%) reported relaxation and meditation techniques to be beneficial (71% in VR, 47% in video). Some of the students commented that “meditation would calm me down,” “it would reduce stress levels,” “it would help me to relax,” and “it would help me to focus.” Forty-two percent of the students (29% in VR, 16 53% in video) did not think the relaxation and meditation techniques would be helpful. Some students said, “I don’t believe in meditation, I believe in studying hard,” “I think it’s the knowledge that matters,” “I think meditation would not help me in any way.” In other words, these students thought that the level of preparation for the test would determine their test scores.

As an extension to the previous question, we asked: “Do you think that this meditation helped you get better scores on the exam?” The majority (60%) of the students said it had (70% in VR, 45% in video). Forty percent of the students felt that meditation did not help them get better scores on the exam. A follow-up question was: “Would you like to do VR or video meditation before an exam again?” The majority (59%) of the students expressed willingness to do so (58% in VR, 58% in video), 27% answered “maybe” (32% in VR, 23% in video), and 13% expressed no interest (9% in VR, 16% in video).

We also wondered about students’ perceptions of the meditation exercises. We asked: “How did you like this meditation?” The response rate was 50%. The majority of the respondents (61%) reported appreciating the meditation (95% in VR, 100% in video). One student in the VR group reported liking the meditation only a little. In addition, we asked: “How immersive was the meditation?” Only half of the students responded. Twenty-four percent of the respondents reported they were very or completely immersed (35% in VR, 3% in video), 13% felt they were immersed just on an average level, 8% felt just a little immersion, and 3% did not feel immersed at all.

The last question targeted only the VR group: “Do you see any positive sides of using VR meditation?” Of the 30 respondents, the majority (70%) saw positive sides of using VR in meditation and the remainder (30%) expressed no opinion. Out of 15 comments given, 66% contained vocabulary such as “relaxing,” “stress-reliever,” suggesting that the VR meditation could relieve stress before an exam. Some specific examples of comments were “VR meditation can relieve the stress” and “you can get relaxed.” The minority of the comments revealed indifference to the VR meditation.

Overall, the majority of students reported perceiving both of the meditation techniques favorably. They expressed increased immersion and beneficial aspects of both types of practice (with a preference for VR). However, these calculations are based only on descriptive statistics so cannot be treated as robust data.

## Discussion

The goal of the study was to examine the differences between the impact of VR- and video-based meditation on college students’ final exam performance. We further explored students’ perceptions of the two types of meditation. Drawing on research by Navarro-Haro et al. (2017), and especially by Kosunen et al. (2016), we hypothesized that VR meditation would be more effective than video meditation. That hypothesis needed to be tested as our study was conducted in an academic setting, and we measured the impact using students’ test scores, not reduction in anxiety levels [as done, for example, by Kosunen et al. (2016)].

Our results showed that students who were exposed to the VR-based meditation benefited from this relaxation technique more than the students who were exposed to the video-based meditation. On one hand, this finding is interesting because some students’ comments in the post-survey expressed a certain level of skepticism toward meditation in general (e.g., “I think meditation would not help me in any way”). Yet, our results suggest that the VR meditation was effective enough to aid the students. This finding allows us to speculate that the reduced anxiety levels allow for better concentration, as found in Mata (2012) and Ching et al. (2015), and can translate into a better exam performance.

On the other hand, our finding is not surprising for two reasons. First, VR is highly immersive (Gruber & Kaplan-Rakowski, 2020; Makransky et al., 2019) and, just like other immersive media, VR can “stimulate physical senses to the point where we experience psychological immersion” (Kaplan-Rakowski & Meseberg, 2019, p. 144). In our experiment, the students immersed in the VR meditation could have had “an impression that it is real and that they are present in this environment” (Kaplan-Rakowski & Meseberg, 2019, p. 144); that is, they could have thought they were in the forest where the meditation took place.

From the multimedia point of view, video and VR have similar audio, but the visual aspect is different. That is, students who experienced video-based meditation, using the desktop monitor, were limited to one view only, while meditation experienced in VR offered 360° visualizations. Students who practiced VR-based meditation thus were surrounded by the forest, not just viewing it. Being a part of the scene allowed for a deeper immersion, facilitating the elicitation of a deeper meditative state. Consequently, this type of meditation allowed students to proceed with exam taking in a relaxed state.

Second, using VR involves wearing a headset, simultaneously isolating individuals from the real world. Such isolation minimizes real-world distractions, allowing subjects to focus on meditation. By contrast, students experiencing video-based meditation may have had more distractions as the space between the monitor and the students’ eyes is large enough that anything happening in that space (e.g., a fly passing by, an extra monitor, the keyboard) may draw attention, not allowing them to focus entirely on the content (in our case, the meditation) being displayed.

The students’ answers to our exploratory questions in the follow-up survey showed a general interest in the use of meditation before exams. Students seemed to have a mostly favorable view of both types of meditation, with a preference for VR. Reporting a positive attitude toward VR technology is a common finding (see, for example, Kaplan-Rakowski & Wojdynski, 2018), given that VR technology was novel to most students. In fact, for several decades now, scholars have pointed out that novelty may lead to the mistaken belief that learning is attributed to a particular technology (e.g., Clark, 1983) and that technology can be VR as well (Huang, 2020).

### Practical implications for educational settings

This study highlights the importance of relaxation techniques in classroom settings, especially when students are likely to experience higher levels of anxiety, as is often the case before or during high-stake tasks. The study used the context of a final exam, but practicing meditation could easily be extended to other typically stressful situations, including public speaking or class presentations.

Incorporating meditation techniques into a school curriculum is a practical way to improve students’ well-being, wellness, and performance because of their relaxing and calming effects (Ching et al., 2015; Mata, 2012). The implementation of meditation practices can be done, for example, by building and incorporating specific meditation modules in learning management systems that students could access on a regular (e.g., daily, weekly) basis. Instructors could either assign or encourage using these meditation modules. The crucial point is offering explicit ways to access meditation modules as students are more likely to take advantage of relaxation techniques when they are easily available, that is, they do not have to be searched for.

While our study was conducted in a traditional classroom setting, its implications can largely extend to meditation practices outside the classroom, with students meditating at their leisure (either at home or on campus). Historically, college campuses have incorporated spaces for spiritual retreats (e.g., chapels) and for relaxation or entertainment (e.g., gaming lounges), or both. This trend can be extended by the establishment of specially designated quiet spaces. Such calming spaces could be useful additions to support students’ cognitive, emotional, or even spiritual needs.

Providing emotional and mental support using positive technology [e.g., artificial intelligence, Dhimolea et al. (2021); mHealth, Riva et al. (2020)] has already been taking place. Therefore, extending this support by promoting VR-based meditation to students would be a natural step forward. Kaplan-Rakowski (2020) emphasized the importance of creating “a learning environment that encourages mind and body balance, such as by the implementation of wellness techniques (e.g., meditation, yoga, exercise) in daily instruction.” (p. 4). Our study provides educational technology and development stakeholders with a clear direction that investing in VR meditation stations in school settings would be beneficial.

### Limitations and future research

This study presents an initial step into research on the implementation of VR-based meditation in academic settings with the intention of promoting reduced anxiety levels and students’ wellness. One of its limitations was that it presented data on students’ self-reported anxiety levels. Future studies should complement subjective data with objective anxiety biometrics. Such measures could be done using wearables such as Empatica 4, with electrodermal activity and heart rate readings reflecting anxiety levels (Gabory & Chollet, 2020; Gjoreski et al., 2016; Gruber & Kaplan-Rakowski, forthcoming). Alternatively, anxiety levels could be measured using salivary cortisol swabs (e.g., Hek et al., 2013; Thrasher, 2021) or with an electroencephalogram (Kosunen et al., 2016).

Another limitation was that the students in our study were exposed to meditation only on one occasion. Even though Tang et al. (2007) pointed out that short-term meditation is becoming increasingly popular without compromising its effectiveness, exposing students to meditation on a regular basis would be more revealing. Besides creating the opportunity to collect longitudinal data, another benefit of such an approach would be eliminating the risk of the novelty effect that is commonly associated with new users of VR (Huang, 2020).

Our study took place in a controlled setting in a computer laboratory. The instructor established the setting, ensuring that the students stayed on task and stuck to the timing of the exam and research procedures. We know from previous research that, due to the immersive nature of VR, it may stimulate Csikszentmihalyi’s “flow” (Bodzin et al., 2020). It is likely that students in noncontrolled circumstances would have taken more time to meditate, which could have had a different effect than what we found. Our meditation exercise lasted 15 min [similar to Mata (2012) and Shamekhi and Bickmore (2015)]. If the meditation had lasted longer, the video meditation could have allowed the students to experience a deeper immersion, thus diminishing the difference between the effect of VR and video meditation. Follow-up studies should investigate an optimal length to obtain benefits without compromising the effectiveness and efficiency of the meditation.

Our sample was based in Poland, where cumulative grading on assignments is often not the case. High-stakes exams tend to be stressful as student careers and futures may depend on performance on one day of a semester. Research done in other countries, in other cultural contexts, and using other technologies and media may yield different findings.

## Conclusions

Meditation can help people reach relaxing states. Incorporating technology into meditation, including video and VR, has also been known to facilitate the meditation practice. Prior to this study, which platform (VR or video) could be more beneficial for students before their final exams was unclear. This study contributed to the body of knowledge by providing early evidence that VR technology may allow learners to better immerse themselves into meditative scenes, consequently eliciting more relaxed states and improved performance. Our evidence carves a pathway to research into positive technology in educational settings. Finally, meditation techniques are not an alternative to exam preparation. Instead, they are potential means for students to relax before events that are typically associated with elevated levels of stress, such as exams.

## Data Availability

Data and materials can be accessed by contacting the lead author.

## Abbreviations

VR: Virtual reality
